# Luteolin Protects Pheochromocytoma (PC-12) Cells against A*β*_25-35_-Induced Cell Apoptosis through the ER/ERK/MAPK Signalling Pathway

**DOI:** 10.1155/2020/2861978

**Published:** 2020-11-30

**Authors:** Han-Rui Wang, Si-Ying Pei, Dong-Xu Fan, Yan-Hui Liu, Xiao-Feng Pan, Fan-Xu Song, Shu-Hua Deng, Hong-Bin Qiu, Ning Zhang

**Affiliations:** ^1^College of Basic Medicine, Jiamusi University, Jiamusi, Heilongjiang, China; ^2^Department of Vascular Surgery, The First Affiliated Hospital of Jiamusi University, Jiamusi, Heilongjiang, China; ^3^Department of Nursing, Jiamusi Central Hospital of Heilongjiang Province, Jiamusi, Heilongjiang, China; ^4^Department of Pediatric Neurology, The Third Affiliated Hospital of Jiamusi University, Jiamusi, Heilongjiang, China; ^5^College of Public Health, Jiamusi University, Jiamusi, Heilongjiang, China; ^6^College of Pharmacy, Jiamusi College, Key Laboratory of Chinese Materia Medica (Ministry of Education), Heilongjiang University of Chinese Medicine, Harbin, Heilongjiang, China

## Abstract

The regulatory effect of luteolin on the progression of Alzheimer's disease (AD) remains unclear from the perspective of apoptosis. The present study aimed to investigate the protective effects of luteolin against A*β*_25-35_-induced cell apoptosis in pheochromocytoma (PC-12) cells. A*β*_25-35_ was used to induce an in vitro model of AD. Estradiol was used as a positive control. The PC-12 cells were incubated with luteolin alone or in combination with fulvestrant or U0126. The results showed that luteolin treatment significantly prevents A*β*_25-35_-induced decrease in cell viability and inhibits A*β*_25-35_-induced cell apoptosis. After the addition of fulvestrant and U0126, the apoptosis rate of PC-12 cells increased significantly. In addition, luteolin treatment significantly upregulated the expression of Bcl-2 and downregulated the expression of Bax and caspase-3, whereas fulvestrant and U0126 partially reversed the effects of luteolin. Moreover, luteolin treatment upregulated the expression of ER*β* and p-ERK1/2, whereas fulvestrant blocked the expression of p-ERK1/2. The study showed that luteolin could activate the ER/ERK/MAPK signalling pathway to protect PC-12 cells against A*β*_25-35_-induced cell apoptosis via selectively acting on ER*β*. Thus, luteolin may be considered as a potential novel therapeutic strategy for AD.

## 1. Introduction

Alzheimer's disease (AD), a chronic progressive neurodegenerative disease, is characterized by degeneration and loss of neurons in the brain, which usually begin with memory loss simultaneously [1]. Most cases of AD are sporadic, with age being the greatest risk factor [2]. It is estimated that there are about 40 millions of people suffering from AD worldwide [3]. Many studies have reported that the deposition of amyloid-*β* (A*β*) fibrils is one of the causes of AD, especially the accumulation of soluble oligomers of A*β*_25-35_ [4, 5]. Therefore, A*β*_25-35_ is usually used to induce an in vitro model of AD [6]. A*β*_25-35_ clusters around neurons, which not only has a direct toxic effect on the neurons but also enhances the sensitivity of neurons to oxidative stress and free radicals, leading to neuron apoptosis [1, 7]. Therefore, the inhibition of A*β*_25-35_-induced cell apoptosis may provide a promising approach for the prevention and treatment of AD.

Flavonoids are a class of about 4000 naturally occurring compounds [8]. It has been reported that flavonoids exhibit numerous biological effects, including antioxidative, anti-inflammatory, and anticancer activity [9, 10]. Luteolin is a kind of flavonoid that is found widely in edible plants such as carrot, broccoli, and *Perilla* leaf [11]. Previous study showed that luteolin improves cognitive dysfunction in AD flies [12]. In addition, study has found that the combination of L-theanine and luteolin may prevent AD-like symptoms, possibly by improving norepinephrine metabolisms and hippocampal insulin signalling and decreasing neuroinflammation [13]. Combining these published evidence, luteolin may be used as a possible therapeutic agent in the treatment of AD. Moreover, it has been shown that luteolin can induce apoptosis of human myeloid leukemia cells, but how luteolin causes this effect has not been definitely elucidated [14]. Most importantly, the regulatory effect of luteolin on the progression of AD remains unclear from the perspective of apoptosis. Thus, the present study aimed to investigate whether luteolin had protective effects against A*β*_25-35_-induced cell apoptosis in PC-12 cells and explored the possible molecular mechanisms for the first time.

## 2. Materials and Methods

### 2.1. Cell Culture and Experiment Design

Rat adrenal PC-12 cell line was purchased from the cell bank of Chinese Academy of Sciences. PC-12 cells were grown in the DMEM complete culture medium (Hyclone, USA) containing 10% fetal bovine serum and 1% double antibody (100 U/mL penicillin and 100 *μ*g/ml streptomycin) in an incubator at 37°C with a 5% CO_2_ atmosphere. The cells in the logarithmic growth phase were selected for subsequent experiments.

The PC-12 cells were divided into 6 groups: (1) blank group: supplemented with 200 *µ*L DMEM; (2) model group: supplemented with 200 *µ*L DMEM for 4 h and then coincubated with 20 *μ*mol/L A*β*_25-35_ [15] (Wuhan Boster Biological Technology Co., Ltd., China) for another 24 h; (3) estradiol group: supplemented with different concentrations (10, 1, 10^−1^, 10^−2^, 10^−3^, 10^−4^, 10^−5^, and 10^−6^ *μ*mol/L) of estradiol for 4 h and then coincubated with 20 *μ*mol/L A*β*_25-35_ for another 24 h; (4) luteolin group: supplemented with different concentrations (10, 1, 10^−1^, 10^−2^, 10^−3^, 10^−4^, 10^−5^, and 10^−6^ *μ*mol/L) of luteolin for 4 h and then coincubated with 20 *μ*mol/L A*β*_25-35_ for another 24 h; (5) luteolin plus fulvestrant group: pretreated with 1 *μ*mol/L fulvestrant [16] (Selleck Chemicals LLC, Houston, TX) for 1 h, supplemented with luteolin for 4 h, and then coincubated with 20 *μ*mol/L A*β*_25–35_ for another 24 h; and (6) luteolin plus U0126 group: pretreated with 10 *μ*mol/L U0126 [17] (Selleck Chemicals LLC, Houston, TX) for 1 h, supplemented with luteolin for 4 h, and then coincubated with 20 *μ*mol/L A*β*_25-35_ for another 24 h. Before adding the A*β*_25-35_ peptide solution to PC-12 cells, it had been incubated at 37°C for 24 h to produce the conformation of fibril or aggregation.

### 2.2. Cell Viability Assay

Cell viability was estimated by the 3-(4, 5-dimethylthiazol-2-yl)-2, 5-diphenyltetrazolium bromide (MTT) assay. In brief, PC-12 cells (3500 cells/well) were seeded into 96-well plates in 200 *µ*L medium and incubated at 37°C for 24 h. Then, the PC-12 cells were exposed to different concentrations (10, 1, 10^−1^, 10^−2^, 10^−3^, 10^−4^, 10^−5^, and 10^−6^ *μ*mol/L) of luteolin or estradiol for 4 h and then coincubated with 20 *μ*mol/L A*β*_25-35_ for another 24 h. After incubation, 20 *µ*L MTT (5 mg/mL) was added to each well. After the cells were incubated for 4 h, 200 *μ*L DMSO was added. The formazan absorbance at 570 nm was measured using the microplate reader (MK3, Thermoelectric, China). The cell viability was expressed as the percentage of the absorbance of treated cells relative to the absorbance of control cells.

### 2.3. Apoptosis Assay by Flow Cytometry

Apoptosis was measured using the Annexin V-FITC/PI double staining kit according to the manufacturer's instruction. In brief, the PC-12 cells were trypsinized, washed with phosphate-buffered saline (PBS), and then resuspended in 400 *μ*L binding buffer. After the cells were incubated in the dark at room temperature for 15 min, 5 *µ*L of Annexin V-FITC and 10 *µ*L of PI were added into cells for flow cytometric analysis.

### 2.4. Quantitative Real-Time PCR (qRT-PCR)

Total RNA was isolated using the TRIzol reagent (Invitrogen, USA). cDNA of *Bcl-2*, *Bax*, and *caspase-3* was prepared using the reverse transcription kit (Sangon, China). *β-*Actin was used as the endogenous control to calculate the relative RNA levels. Data were calculated by the comparative cycle threshold (CT) (2^−ΔΔCT^) method. The PCR primer sequences were as follows:

1. *Bcl-*2 forward: 5′-AGCCTGAGAGCAACCGAAC-3′ and reverse: 5′-AGCGACGAGAGAAGTCATCC-3′
2. *Bax* forward: 5′-TGCTACAGGGTTTCATCCAG-3′ and reverse: 5′-ATGTTGTTGTCCAGTTCATCG-3′
3. *Caspase-3* forward: 5′-TGACTGGAAAGCCGAAACT-3′ and reverse: 5′-TGACTGGAAAGCCGAAACT-3′
4. *β*-Actin forward: 5′-TGTCACCAACTGGGACGATA-3′ and reverse: 5′-GGGGTGTTGAAGGTCTCAAA-3′

### 2.5. Western Blot

Western blot was performed to detect the effects of luteolin on the expression of apoptosis factors. The total protein was extracted with RIPA lysate, and the protein was quantified and denatured to obtain a protein sample. Equal amounts of proteins were subjected to sodium dodecyl sulfate-polyacrylamide gel and then transferred to polyvinylidene fluoride (PVDF) membranes (Millipore, USA). The PVDF membrane was blocked in 5% nonfat milk for 2 h at room temperature and subsequently incubated at 4°C overnight with antibodies against Bcl-2, Bax, caspase-3, ER*β*, or *β*-actin (Beijing Boosen Biological Technology Co., Ltd., China). Next, the PVDF membrane was incubated with corresponding horseradish peroxidase-conjugated (HRP)-linked secondary IgG antibodies (Wuhan Boster Biological Technology Co., Ltd., China) for 2 h at 37°C. The enhanced chemiluminescence (ECL) kit (Beyotime, Shanghai, China) was used to detect immunoreactive bands, and a lane ID gel analysis system was used to analyse the grey values.

### 2.6. Statistical Analysis

All statistical analyses were performed by using SPSS version 18.0 (SPSS Institute, IL, USA). Results from each experiment were expressed as the mean ± standard deviation of three separate experiments. The comparison of different groups was performed by one-way analysis of variance (ANOVA) followed by least significant difference- (LSD-) *t*-test or Tukey's test for multiple comparisons. Statistical significance was set at *p* < 0.05.

## 3. Results

### 3.1. Luteolin Improved the Viability of PC-12 Cells Damaged by A*β*_25-35_

**Figure 1:**
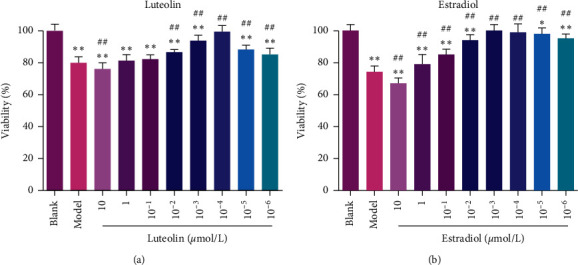
Effects of luteolin on viability in PC-12 cells injured by A*β*_25-35_. (a) Quantitative assessment of cell viability after treatment with different concentrations (10, 1, 10^−1^, 10^−2^, 10^−3^, 10^−4^, 10^−5^, and 10^−6^ *μ*mol/L) of luteolin by MTT. (b) Quantitative assessment of cell viability after treatment with different concentrations (10, 1, 10^−1^, 10^−2^, 10^−3^, 10^−4^, 10^−5^, and 10^−6^ *μ*mol/L) of estradiol by MTT. Data were presented as the mean ± standard deviation (*n* = 6). ^*∗*^*p* < 0.05 and ^*∗∗*^*p* < 0.01, compared with the blank group; ^##^*p* < 0.01, compared with the model group.

In order to verify the cytotoxic and neuroprotective effects of luteolin for PC-12 cells, cell viability was performed by the MTT assay. As shown in Figure 1(a), exposure to A*β*_25-35_ (model group) significantly decreased the viability of PC-12 cells compared with the blank group (*p* < 0.05). Luteolin was shown to protect PC-12 cells from A*β*_25-35_-related toxicity (except 10 *μ*mol/L concentration). Among the eight concentrations, lueolin at the concentration of 10^−4^ *μ*mol/L could significantly prevent A*β*_25-35_-induced decrease in cell viability. Therefore, 10^−4^ *μ*mol/L luteolin was used for subsequent studies.

In addition, estradiol was used as a positive control (Figure 1(b)). Compared with the model group, the cell viability in the estradiol group was significantly increased (all *p* < 0.05) at different concentrations (1, 10^−1^, 10^−2^, 10^−3^, 10^−4^, 10^−5^, and 10^−6^ *μ*mol/L). Treatment with 10^−3^ *μ*mol/L estradiol could significantly prevent A*β*_25-35_-induced decrease in cell viability. Therefore, 10^−3^ *μ*mol/L estradiol was used for subsequent studies.

### 3.2. Luteolin Inhibited A*β*_25-35_-Induced Cell Apoptosis

**Figure 2:**
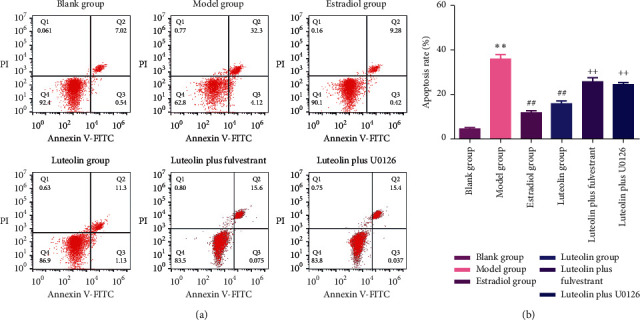
Luteolin inhibited A*β*_25-35_-induced cell apoptosis. (a) Flow cytometry was used to detect the apoptosis of PC-12 cells. (b) Quantitative assessment of the apoptosis rate. Data were presented as the mean ± standard deviation. ^*∗∗*^*p* < 0.01, compared with the blank group; ^##^*p* < 0.01, compared with the model group; ^++^*p* < 0.01, compared with the luteolin group.

Flow cytometry was performed to detect the effect of luteolin on A*β*_25-35_-induced cell apoptosis. The results showed that estradiol and luteolin could inhibit A*β*_25-35_-induced cell apoptosis (Figure 2). After the addition of fulvestrant (luteolin plus fulvestrant group) and U0126 (luteolin plus U0126 group), the apoptosis rate of PC-12 cells increased significantly (*p* < 0.05). These results indicated that luteolin might inhibit A*β*_25-35_-induced cell apoptosis through ER/ERK/MAPK signalling pathways.

**Figure 3:**
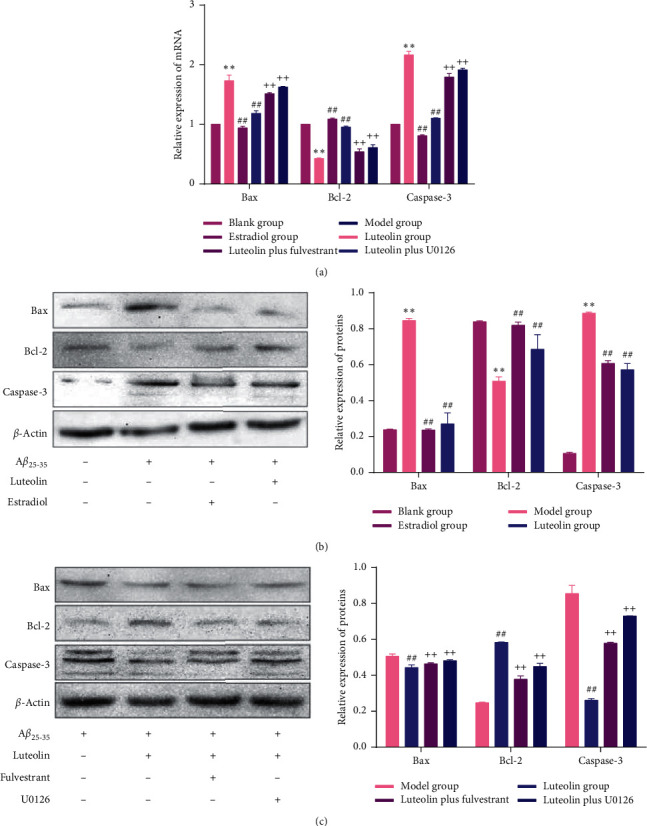
Expression of genes and proteins related to apoptosis. (a) qRT-PCR analysis of Bcl-2, Bax, and caspase-3 mRNA in each group. (b) Western blot analysis of Bcl-2, Bax, and caspase-3 expression in each group. (c) Western blot analysis of Bcl-2, Bax, and caspase-3 expression after the addition of fulvestrant and U0126. Data were presented as the mean ± standard deviation. ^*∗∗*^*p* < 0.01, compared with the blank group; ^##^*p* < 0.01, compared with the model group; ^++^*p* < 0.01, compared with the luteolin group.

To further verify the effect of luteolin on A*β*_25-35_-induced cell apoptosis, we detected the expression of genes and proteins related to cell apoptosis (Figure 3). The results showed that the expressions of Bax and caspase-3 (apoptosis promoters) at mRNA and protein levels in the model group were significantly higher than those in the blank group (all *p* < 0.01). In addition, the expressions of Bcl-2 (apoptosis inhibitor) at mRNA and protein levels in the model group were significantly lower than those in the blank group (all *p* < 0.01). Luteolin and estradiol significantly downregulated the expression of Bax and caspase-3 and upregulated the expression of Bcl-2 (Figures 3(a) and 3(b)), whereas fulvestrant and U0126 partially reversed the effects of luteolin (all *p* < 0.01, Figures 3(a) and 3(c)). These results also indicated that ER/ERK/MAPK signalling pathways might be involved in the protective role of luteolin against A*β*_25-35_-induced cell apoptosis.

### 3.3. Luteolin Inhibited A*β*_25-35_-Induced Cell Apoptosis through the ER/ERK/MAPK Signalling Pathway

**Figure 4:**
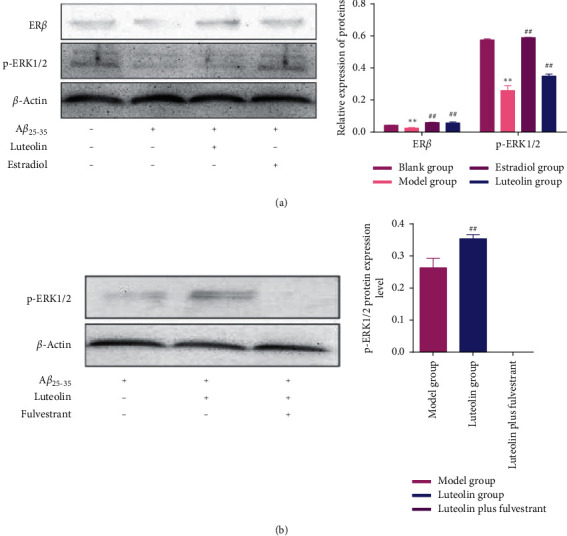
Luteolin treatment upregulated the expression of p-ERK1/2. (a) Western blot analysis of ER*β* and p-ERK1/2 expression in each group. (b) Fulvestrant blocked the expression of p-ERK1/2. Data were presented as the mean ± standard deviation. ^*∗∗*^*p* < 0.01, compared with the blank group; ^##^*p* < 0.01, compared with the model group.

To further verify our hypothesis, we detected the expression of proteins related to the ER/ERK/MAPK signalling pathway. As shown in Figure 4(a), the expression level of ER*β* and phosphorylated ERK1/2 (p-ERK1/2) in the model group was significantly lower than that in the blank group (*p* < 0.01). No ER*α* proteins were detected in any group. In addition, luteolin and estradiol significantly upregulated the expression of ER*β* and p-ERK1/2 (Figure 4(a)), whereas fulvestrant blocked the expression of p-ERK1/2 (Figure 4(b)). These results indicated that luteolin could activate the ER/ERK/MAPK signalling pathway to protect PC-12 cells against A*β*_25-35_-induced cell apoptosis via selectively acting on ER*β*.

## 4. Discussion

AD is an age-related progressive neurodegenerative disease which is characterized by loss of learning and memory in the early stages [18]. A*β*_25-35_ deposition is the main histopathological feature of AD, which leads to degeneration and apoptosis of nerve cells, further leading to cognitive dysfunction and memory loss [19, 20]. Thus, the regulation of A*β*_25-35_-induced cell apoptosis to prevent the occurrence of AD may be a promising approach. Previous studies have confirmed that luteolin plays an important role in the process of AD [21, 22]. However, the underlying mechanism has not been well elucidated. In this study, we established an in vitro model of AD and investigated the possible mechanisms of luteolin against A*β*_25-35_-induced cell apoptosis for the first time.

Luteolin, a naturally occurring flavonoid found in edible plants, had been found to have chemopreventive effects against malignant tumors in vivo without toxic side effects [23, 24]. In the present study, we first verified the effect of luteolin on cell viability. The results showed that luteolin could protect PC-12 cells from A*β*_25-35_-related toxicity at different concentrations (10^−2^, 10^−3^, 10^−4^, 10^−5^, and 10^−6^ *μ*mol/L). Surprisingly, we found that luteolin showed a significant toxic side effect at a dose of 10 *μ*mol/L, which reminded us that high doses of luteolin may have potential toxicity. The present study showed that luteolin at the concentration of 10^−4^ *μ*mol/L could significantly prevent A*β*_25-35_-induced decrease in cell viability. Similarly, Guo et al. demonstrated that luteolin could significantly attenuate 6-OHDA-induced PC-12 cell viability loss in a dose-dependent manner [25]. Besides, we also detected the effect of luteolin on A*β*_25-35_-induced cell apoptosis. The study performed by Guo et al. had indicated that the increased apoptotic rate induced by 6-OHDA in the PC-12 cell could be significantly inhibited by luteolin (12.5 or 50 *µ*M) [25]. In the present study, we found that luteolin could inhibit A*β*_25-35_-induced cell apoptosis. These results indicated that luteolin could protect PC-12 cells against A*β*_25-35_-related toxicity.

Next, we further investigated the possible mechanism of luteolin against A*β*_25-35_-induced cell apoptosis. Previous studies had demonstrated that A*β*-induced apoptosis could be regulated by triggering an intracellular apoptotic cascade, including the activation of caspase-3 and Bax in hippocampus neurons [26, 27]. In the present study, we found that luteolin treatment could significantly upregulate the expression of Bcl-2 and downregulate the expression of Bax and caspase-3. Extracellular signal-regulated kinase 1/2 (ERK1/2) belonged to the mitogen-activated protein kinase (MAPK) family, which plays an important role in the signal cascade and transfers extracellular signals to intracellular targets [28]. Evidence had showed that the bee venom-induced apoptosis in human leukemic cells was through the downregulation of the ERK and Akt signal pathway [29]. The present study showed that the addition of fulvestrant (ER antagonist) and U0126 (ERK inhibitor) partially reversed the effects of luteolin on the apoptosis rate and apoptosis-related genes and proteins. These results indicated that luteolin might inhibit A*β*_25-35_-induced cell apoptosis through ER/ERK/MAPK signalling pathways.

To further verify our hypothesis, we detected the expression of proteins related to the ER/ERK/MAPK signalling pathway. It was known that the phosphorylation of ERK1/2 activated a series of protein signalling cascades that regulated a variety of cellular processes, such as plasticity of neurons, growth, and survival [30]. The present study showed that luteolin significantly upregulated the expression of p-ERK1/2, whereas fulvestrant blocked the expression of p-ERK1/2. Estrogen receptors (ERs) existed in two isoforms: ER-alpha (ER*α*) and ER-beta (ER*β*) [31]. ER*α* was mainly expressed in reproductive tissues, bone, kidney, liver, and white adipose tissue, while the expression of ER*β* was found in the central nervous system, cardiovascular system, male reproductive organs, and the immune system [32]. In the present study, we found that luteolin significantly upregulated the expression of ER*β*, whereas no ER*α* proteins were detected in any group. Several studies had demonstrated the ability of estrogen mediated by the ER to activate the PI3 and MAPK signalling pathway [33]. These results indicated that luteolin could activate the ER/ERK/MAPK signalling pathway to protect PC-12 cells against A*β*_25-35_-induced cell apoptosis via selectively acting on ER*β*.

## 5. Conclusions

The present study further confirmed the effects of luteolin on A*β*_25-35_-induced cell apoptosis. Our studies demonstrated that luteolin could significantly inhibit A*β*_25-35_-induced cell apoptosis, whereas fulvestrant and U0126 partially reversed the effects of luteolin. Additionally, luteolin treatment upregulated the expression of ER*β* and p-ERK1/2, whereas fulvestrant blocked the expression of p-ERK1/2. Taken together, our studies demonstrated that luteolin could activate the ER/ERK/MAPK signalling pathway to protect PC-12 cells against A*β*_25-35_-induced cell apoptosis via selectively acting on ER*β*. Thus, luteolin may be considered as a potential novel therapeutic strategy for AD.

## Data Availability

All data generated or analysed during this study are included in this published article.
